# Fast differential scanning calorimetry to mimic additive manufacturing processing: specific heat capacity analysis of aluminium alloys

**DOI:** 10.1007/s10973-022-11824-4

**Published:** 2022-12-07

**Authors:** Cameron R. Quick, Phillip Dumitraschkewitz, Jürgen E. K. Schawe, Stefan Pogatscher

**Affiliations:** 1 Chair of Non-Ferrous Metallurgy, Montanuniversitaet Leoben, Leoben, Austria; 2 Mettler-Toledo GmbH, Analytical, 8606 Nänikon, Switzerland; 3 Laboratory of Metal Physics and Technology, Department of Materials, ETH Zurich, 8093 Zurich, Switzerland

**Keywords:** Specific heat capacity, Fast differential scanning calorimetry (FDSC), Aluminium alloys, Precipitation, Additive manufacturing (AM)

## Abstract

Eutectic AlSi12, commonly used in casting and in additive manufacturing, is investigated with Fast Differential Scanning Calorimetry to determine the impact of different cooling rates from the liquid state upon the apparent specific heat capacity on subsequent heating. A heat flow correction strategy is developed and refined for the reliable and precise measurement of sample heat flow using chip sensors and assessed by the evaluation of results on pure (99.999%) aluminium. That strategy is then applied to the study of the AlSi12 eutectic alloy, and rate-dependent perturbations in the measured apparent specific heat capacity are discussed in terms of Si supersaturation and precipitation. Several cooling rates were implemented from − 100 to − 30,000 K s^−1^, and subsequent heating ranged from + 1000 to + 30,000 K s^−1^. After rapid cooling, a drop in AlSi12 apparent specific heat capacity is found on heating above ~ 400 °C; even at rates of + 10,000 K s^−1^, a result which has high relevance in metal additive manufacturing where similarly fast temperature cycles are involved. The Literature data, temperature modulated DSC and CALPHAD simulations on the heat capacity of AlSi12 are used to provide comparative context to the results from Fast Differential Scanning Calorimetry.

## Introduction

A well-established technique to measure the thermodynamics and kinetics of phase transformations in various materials is Differential Scanning Calorimetry (DSC). However, the addenda heat capacity and the resulting thermal lag of conventional DSC apparatus limit the scanning rates to several hundred Kelvin per minute [1, 2]. This is too slow for studying the solidification of many technical processes like injection moulding, welding and laser sintering at realistic rates. To increase the applicable scanning rate range for heating and cooling by more than 4 decades, the non-adiabatic chip-based Fast DSC (FDSC) was developed [3–5]. An example which demonstrates the potential of this technique is discussed by Cebe et al. [6, 7]. There, the specific heat capacity of silk fibroin was successfully measured in the melt far above its normal decomposition temperature, since the heating rate of FDSC was high enough to shift decomposition to higher temperatures. For determination of the specific heat capacity, this was coupled with a method for heat loss correction [6, 8–12].

The major advantages of FDSC originate from the high heating and cooling rates possible compared to conventional DSC. These capabilities have fostered particular interest in the polymer and glass communities since the advent of FSC in the nineties [13], as kinetic-based crystallisation effects can be investigated with relative ease over a much broader range of temperature rates [14]. The ability to implement such a broad range of cooling rates means that the impact of thermal treatments can be investigated in great depth and detail. These capabilities also hold many advantages for investigation of metallic systems, whose properties vary due to microstructural or phase content differences brought about either by mechanical or thermal manipulation. FSC provides an opportunity to control a sample’s state through implementation of user defined temperature programmes. Mettler Toledo’s fast scanning calorimeter, the Flash DSC 2+ launched in 2019, covers temperatures from − 100 to + 1000 °C and rates of − 40,000 to + 50,000 K s^−1^ and has great potential to study many materials in all fields of science. Metallic materials have been investigated with FSC in several contexts in materials science, including bulk metallic glasses (BMG) [15–18] and additive manufacturing [19–21] as well as nucleation and crystallisation [22, 23].

The high heating and cooling rates possible when using the Flash DSC 2+ with the MultiSTAR UFH 1 sensors hold some particular relevance for metal additive manufacturing (AM), since the associated processing techniques [e.g. in laser powder bed fusion (LPBF)] implement temperature changes in the tens of thousands of Kelvin per second [24]. Calorimetric measurement at such rates is only possible using FSC. Current approaches for assessment in AM often rely on mechanical testing of printed parts [25, 26] or on metallographic [25] investigations of, for example, single scan tracks [27, 28]. Single scan tracks are produced when the laser melts the powder bed in a line and are then cut, polished and analysed. They require only a small amount of alloy powder to produce whilst still providing insight on the nature of the melt pool and suitable scanning parameters; however, direct insight into thermo-physical properties during the process is rarely given. FSC measurements have already provided some insight into precipitation mechanisms at high undercooling for example in the works by Yang et al. [21] and Zhuravlev et al. [20]. Other contemporary research in AM is based on computational modelling and simulations. For this, heat capacity data are typically implemented with temperature-dependent equilibrium data, as measured via conventional DSC methods, or even as a single fixed value. FSC analysis on the other hand allows direct calorimetric measurement at process-comparable cooling rates and therefore, can provide measurements at highly relevant non-equilibrium conditions.

Recently, the authors reported on experiments and correction methods to determine the heat capacity of pure Pb (99.999%) using the low-temperature MultiSTAR UFS 1 chip sensor [9]. However, the high-temperature UFH 1 chip sensors constitute a redesign with a thinner membrane, smaller heated area and gold instead of aluminium to withstand higher temperatures. This demands a reassessment of optimal measurement parameters. The most obvious of these is the lower sample mass required versus the UFS 1 chip sensors, which helps to achieve much faster heating and cooling rates. Beyond this, according to the user manual for the Flash DSC 2+, UFH 1 sensors tend to have shorter lifespans before breakage, particularly when operating at high temperatures. Experiment design should ideally take this into account to best utilise the chip sensors.

The present work explores a methodology of precise, reproducible heat capacity measurements with the high-temperature MultiSTAR UFH 1 chip sensors, the basis of which revolves around measuring and correcting for systematic heat losses. For the determination of such measurement strategy, pure aluminium is studied. To demonstrate application of this strategy in practice, we study the AlSi12 alloy common in casting and AM. The present work focuses on precise measurements of AlSi12 powder and determination of the effect of different cooling and heating rates upon the measured heat capacity. The results use the fact that the resultant curves are not simply the materials *c*_p_, but the superposition of the heat capacity and any other thermal effects at that moment, to draw conclusions about the material changes involved during rapid processing.

## Experimental

### CALPHAD calculations

The equilibrium heat capacity of AlSi12 was calculated with FactSage 8.0 [29] via the Equilib module and the function builder using the light metal alloy database FTlite 2020 [30].

### Fast differential scanning calorimetry

The FDSC analysis was performed using a Mettler Toledo Flash DSC 2+ equipped with an intracooler on conditioned and corrected MultiSTAR UFH 1 high-temperature sensors under an argon flow of 80 mL min^−1^. The sensor support temperature was set to − 90 °C.

### Materials

Aluminium foil was obtained from Alfa Aesar (Karlsruhe, Germany) at 99.9996% purity and 38 ± 7 µm thickness. AlSi12 powder (aluminium with 12 mass% silicon) was sourced from inspire AG (Zurich, Switzerland).

### FDSC sample preparation

Samples of pure aluminium were prepared from 38 µm foil using a scalpel to cut an appropriately sized piece (~ 50 µm) and positioned using a hair stylus. The sample was then melted and solidified several times to achieve a consistent interface and provides good thermal contact with the sensor. The sample preparation for AlSi12 simply required isolating a single particle of the alloy powder usually used for AM and positioning it on the sensor. A very small quantity of a silicon oil spread on the sensor’s sample area aided in achieving the ideal sample position and helped to stop the sample jumping away during first heating. The silicon oil is vaporised during the first heating programme [31], where the sample is again melted and solidified several times.

### Slow-rate heat flow correction

Building on the work in [9], where the necessity of heat loss correction was demonstrated, two approaches for determining the system’s heat losses was considered. One, the so-called symmetry correction, uses a symmetric heating and cooling programme to evaluate the temperature-dependant heat loss curve. However, this approach is only suitable without the occurrence of irreversible transitions in a sample that could impact the heat flow (e.g. in a super saturated solid solution). Since the present work should not only be suitable for pure metals (i.e. here Al), but also for alloys (i.e. here the eutectic AlSi12), the symmetry approach was rejected in favour of the slow-rate approach for heat loss determination. Equation (1) relates the measured heat flow ($\phi (T)$) to the sample mass ($m$), the specific heat capacity (${c}_{\mathrm{p}}(T)$), the heating rate ($\beta$) and the systematic heat losses (${\phi }_{\mathrm{loss}}(T)$) [10].

1 $$\phi (T)=m\bullet {c}_{\mathrm{p}}(T)\bullet \beta +{\phi }_{\mathrm{loss}}(T)$$

The slow-rate approach approximates the heat flow at a very low rate, e.g. + 1 K s^−1^, to represent the systematic heat losses, turning Eq. (1) into (2) [9, 10].

2 $${\phi }_{\beta =1 {\mathrm{K s}}^{-1}}\left(T\right)\approx {\phi }_{\mathrm{loss}}(T)$$

This approximation is further validated when considering the comparative magnitude of the sample signal in the slow and rapid heating scans. That is, the sample contribution in the 1 K s^−1^ slow scan is 3–4 magnitudes smaller than in the rapid-rate curves [9, 10], causing a negligible error of less than 0.1%.

Having determined the heat loss function, the measured heat flow can then be corrected according to Eq. (3). Dividing by the programmed heating rate and the determined sample mass then yields the heat capacity curve.

3 $$\phi \left(T\right)-{\phi }_{\mathrm{loss}}\left(T\right)=m\bullet {c}_{\mathrm{p}}(T)\bullet \beta$$

### Mass determination for FDSC samples

For the measurements on pure aluminium, the sample mass was determined by the ratio between the melting enthalpy Δ*H* and the specific enthalpy of fusion Δ*h*_fus_:

4 $$\frac{\Delta H}{\Delta {h}_{\text{fus}}}=m$$

The melting enthalpy is determined by integration of the uncorrected heat flow curve during melting, and $\Delta {h}_{\mathrm{fus}}=397 \mathrm{J }{\mathrm{g}}^{-1}$ is the specific enthalpy of fusion of aluminium [32]. The sample mass is 64 ng.

Similarly, Eq. (4) was used to determine the AlSi12 sample mass from the integrated melting peak measured via FDSC, taking the specific enthalpy of fusion ${\Delta h}_{\mathrm{fus}}^{\mathrm{AlSi}12}=$ 560 J g^−1^ [33]. For the two samples measured, the melting peak from an uncorrected heating segment at + 1000 K s^−1^ was integrated and yielded masses of 100 ng and 26 ng.

### Temperature modulated DSC

Temperature Modulated DSC (TMDSC) was introduced by Reading et al. [34]. The goal was to separate “reversing” and “non-reversing” heat flow by superimposing the conventional temperature programme with a periodic (sinusoidal) temperature perturbation. The technical but weak physical definition of the term “reversing heat flow” was subject to some controversy [35–37]. Despite these discussions, TMDSC was used for measurement of the heat capacity [38]. A generalised theory of TMDSC was given 2006 [39]. It was shown that reversing heat flow is the sensible heat flow (driven by external temperature change) for quasi-static conditions. To fulfil such a condition, it was proposed to substitute the single frequency temperature modulation with a frequency spectrum by stochastic modulation [39]. Furthermore, an advanced evaluation procedure was proposed [39, 40]. This modulation technique was commercialised as TOPEM by Mettler Toledo.

The TOPEM measurements were performed in a temperature range between 25 and 550 °C with an underlying heating rate of 1 K min^−1^. Sapphire measurements are used for calibrating the heat capacity. To avoid eventual reactions with the crucible, 30 μl alox crucibles with lid (typical mass: 150 mg) are used. The maximum mass difference between the pans of the sample, sapphire and reference was about ± 0.5 mg. The related heat capacity error was not compensated.

The modulation function was defined by the minimum and maximum switching time of 50 and 60 s and a step height of ± 1 K. The sampling distance was 0.1 s. The measurements were performed using a DSC 1 from Mettler Toledo equipped with FRS 6 sensor. The mass of the studied AlSi12 powder sample was 27.5 mg. The high density and high thermal conductivity of metals mean masses of 30–60 mg are appropriate for decent signal strength without considerable thermal lag [41]. The following evaluation parameters are used: Evacuation Window 400 s, Sample Response Parameter 2, Instrumental Response Parameter 60.

### Apparent heat capacity

The heat flow into a sample during a DSC measurement contains two components, the sensible heat flow, *ϕ*_s_ and the latent heat flow, *ϕ*_l_:

5 $$\phi = \phi_{{\text{s}}} + \phi_{{\text{l}}} = m c_{{\text{p}}} \beta + m \Delta h_{{\text{l}}} \frac{{{\text{d}}\xi }}{{{\text{d}}t}}$$

where Δ*h*_l_ is the specific enthalpy of a latent thermal process, and *ξ* is the internal order parameter related to the latent process. The sensible heat flow is driven by the external temperature change *β* and is proportional to *c*_p_. The latent heat is driven by the change of the internal order parameter *ξ* and is proportional to Δ*h*_l_.

Equation (5) is used to define the apparent specific heat capacity that includes sensible and latent properties:

6 $$c_{{{\text{p}},{\text{a}}}} = \frac{\phi }{m \beta } = c_{{\text{p}}} + \Delta h_{{\text{l}}} \frac{{{\text{d}}\xi }}{{{\text{d}}T}}$$

In the case of TOPEM measurements, the sensible and latent heat flow can be assumed to be the reversing and non-reversing heat flow, respectively.

## Results

### *c*_p_ determination of pure aluminium

**Fig. 1:**
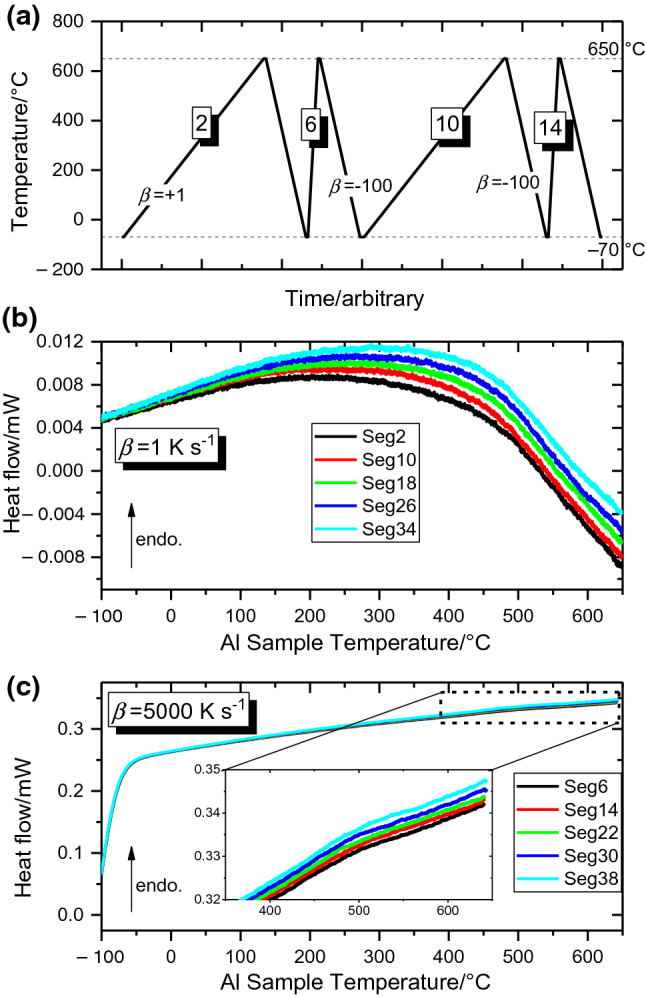
Measurements on pure Al provide justification for the structure of the implemented temperature programmes. Two repeat units of the implemented temperature programme are shown in (**a**), whilst the heat flow measured on heating at + 1 K s^−1^ and + 5000 K s^−1^ are shown in (**b**) and (**c**). Readers should note that the absolute value of the measured heat flow changes with subsequent heat scans. As such, the slow-rate heat loss correction curve should be repeatedly re-measured for each analysed heating scan

In order to develop a measurement strategy for AlSi12, preliminary investigations were performed on pure Al due to its well-known thermal properties and easy interpretation since any non-equilibrium effects are negligible within the expected resolution. Figure 1 shows heat flow curves during heating measured by FDSC, and the implemented temperature programme was cycled 5 times (Fig. 1a shows two cycles), and the measured heat flow for the slow heating segment (+ 1 K s^−1^) and the faster heating segment (+ 5000 K s^−1^) are displayed in (b) and (c), respectively. The plots clearly show an incremental increase in the measured heat flow with each iteration: approximately 4–5 µW at 500 °C after 5 measurement cycles. This is significantly higher than the expected drift of the sensor (< 5 µW per hour according to the user manual). In addition to sensor drift, this behaviour can be caused by the change in thermal contact. For the determination of the heat capacity, the effects of these changes should be minimised. This can be done by: (i) programming short isothermal segments; (ii) reducing the number of superfluous measurement runs; and (iii) by using the lowest maximum temperature feasible to achieve the desired results. Moreover, when the heat loss correction curve is measured as near as possible to the heat scan intended for analysis, we can surmise that the impact of such gradual changes has been minimised. It is these considerations that influenced the design of the used temperature programmes, as exemplified in Fig. 1a. The slow-rate heat step (label “2”) records the heat flow correction curve; after an 0.1 s isothermal step, the sample is cooled at a rate which defines the system’s microstructure, and after another 0.1 s, the sample is heated at the rate intended for analysis (label “6”). Cycling this time–temperature programme, and implementing the slow-rate correction to the heat flow only adjacent measurement segments, provides a reliable recipe for precise heat flow measurements. By changing the implemented heating and cooling rates, the impact of such conditions on the measured heat flow may be mapped out and understood, the results of which can be seen in Fig. 3 for that of pure aluminium, and continued in later figures to that of AlSi12. A final note on the temperature programmes implemented for pure aluminium is that, aside from the initial melting-solidification programmes to achieve good sample sensor contact, the sample remained in the solid state; that is, the heat capacity measurement programmes’ maximum temperature was 10 degrees below the sample melting point. The pure Al melting point onset was used to correct the curves’ recorded temperature.

**Fig. 2:**
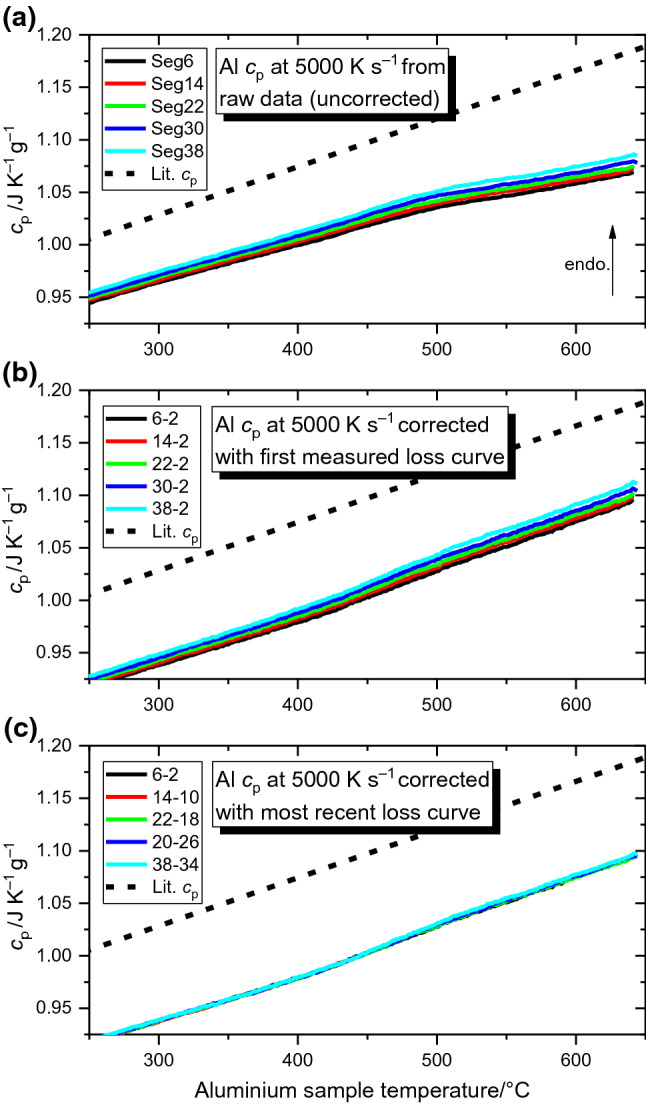
Specific heat capacity curves for pure aluminium using the data from Fig. 1. For visual clarity, the 250–650 °C range is shown, whilst Fig. 3 shows the complete corrected *c*_p_ curves. Panel **a** shows the heat capacity determined on heating at + 5000 K s^−1^ with no heat flow correction, **b** shows the heat capacity determined when subtracting only the first + 1 K s^−1^ segment to account for heat losses, whilst **c** shows the heat capacity determined when the heat flow for each + 5000 K s^−1^ segment is corrected by subtracting the heat flow of the most recent + 1 K s^−1^ segment

**Fig. 3:**
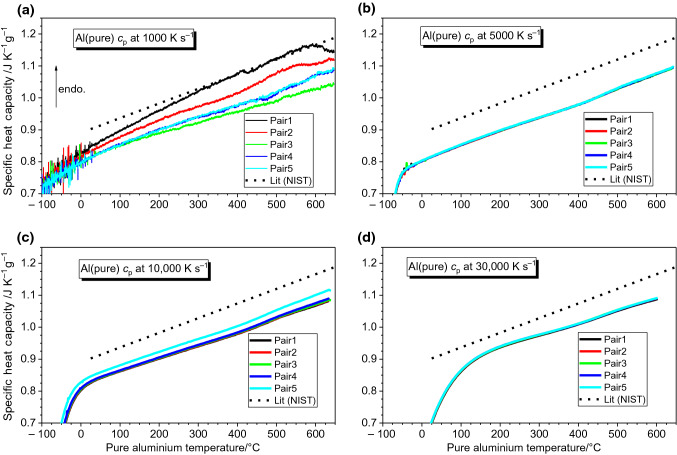
Specific heat capacity curves for pure Al determined from slow-rate corrected heat flow on heating at **a** 1000 K s^−1^; **b** 5000 K s^−1^; **c** 10,000 K s^−1^ and **d** 30,000 K s^−1^. The mass was determined as 64 ng by integrating the sample’s melting peak and curve temperature has been adjusted to match the literature melting temperature for Al [32]

To further examine the influence of the heat flow drift on the heat capacity determination, heat capacity curves are determined from the data in Fig. 1b, c in three ways and presented in Fig. 2: (a) without heat loss corrections; (b) subtracting only the first + 1 K s^−1^ heat loss curve; and c) always subtracting the most recent + 1 K s^−1^ heat loss curve. For clarity in comparison, the 250–650 °C range is shown, whilst the complete curves are included in Fig. 3. With no heat flow corrections as in Fig. 2a, the determined heat capacity curves are not parallel to the literature values [32]. Additionally, there is a perturbation above ~ 500 °C which further differs the results from the literature data. Applying a correction to the heat flow by subtracting the heat flow of the initial + 1 K s^−1^ segment (Fig. 2b) both reduces these perturbations and brings the curves parallel to the literature data. Both (a) and (b) however still exhibit the incremental changes observed in the raw data of Fig. 1 with successive cycles. Correcting the heat flow by instead subtracting the most recent + 1 K s^−1^ heat loss measurement from each + 5000 K s^−1^ segment eliminates these incremental changes, with all *c*_p_ curves lying on top of one another. The correction method of Fig. 2c is then employed for all further *c*_p_ measurements.

Employing the outlined measurement strategy of slow-rate heat flow correction, the heat capacity of pure aluminium is measured on heating at rates of 1000, 5000, 10,000 and 30,000 K s^−1^ and is presented in Fig. 3a–d, respectively. As expected, the noise of the heat capacity signal decreases with increasing the scanning rate due to the improved signal-to-noise ratio. Due to the thermal lag of the system, the temperature at which the measuring system reaches steady state conditions increases with increasing scanning rate. The intermediate rates at + 5000 and + 10,000 K s^−1^ occupy a fair middle ground with excellent reproducibility, meaning the inherent noise is simple to reduce with curve averaging. Since the measured curves are all ~ 10% lower than the literature values, the determined mass of 64 ng is likely ~ 10% higher than the true value for this sample, though it was found to fluctuate up to 2 or 3 ng.

### Application to AlSi12 powder for additive manufacturing

**Fig. 4:**
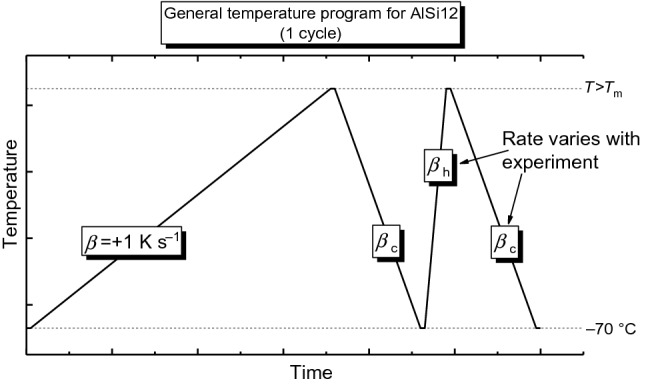
The general temperature programme followed for the experiments on AlSi12. The + 1 K s^−1^ heat segment is subtracted from the faster (*β*_h_) heating segment to provide the slow-rate corrected heat flow. This figure’s temperature cycle is implemented at least 2 times for each investigated rate, and the slow-rate correction as described in Sect. 3.1 is performed for each cycle. Further repetitions were implemented if inconsistencies were found in the resultant curves. The corrected heat flow curves are then averaged and divided by heating rate, and sample mass to determine the heat capacity curves

Figure 4 shows a schematic of the temperature programmes implemented on the AlSi12 powder and follows the same method as that employed for pure Al. For the measurements on AlSi12, the cooling step was implemented from the liquid state to better mimic AM processing. The relationship between the cooling rate and the alloy’s microstructure can be analysed by means of the subsequent heating measurement. Quantitative heat capacity measurements here allow small differences in the microstructure to be indirectly detected. The maximum temperature was chosen as 800 °C, which ensured complete melting even at the highest heating rate (+ 30,000 K s^−1^). The heating and cooling rates were chosen to evaluate their impact on apparent heat capacity from near-equilibrium to AM-relative rates (+ 1000 K s^−1^ to + 30,000 K s^−1^ and − 100 to − 30,000 K s^−1^). Thermal lag in FSC has been investigated on several metallic systems and is in the range between 0.2 ms and a few milliseconds [18, 42], with the 100 ms being more than enough time to reach thermal equilibrium.

**Fig. 5:**
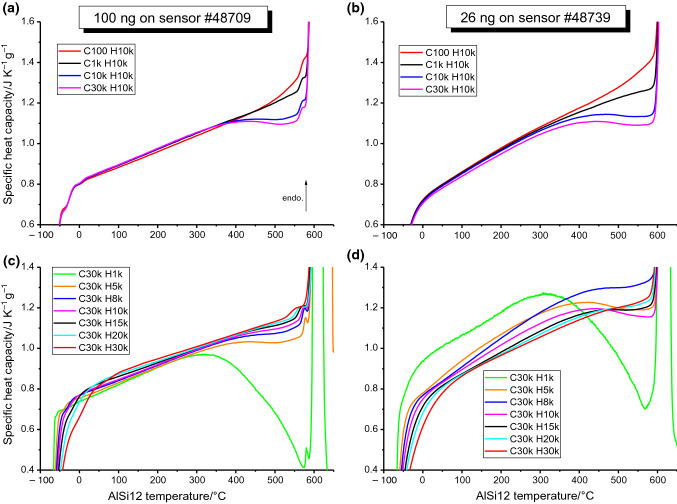
Apparent heat capacity curves on heating 2 samples of AlSi12 with various thermal histories. Panels **a** and **b** compare the measured heat capacity on heating at + 10,000 K s^−1^ after different prior cooling rates for a 100 ng and 26 ng sample, respectively, whilst **c** and **d** compare the measured heat capacity on heating the same respective samples at various rates after a prior cooling of − 30,000 K s^−1^. The distinct drop in apparent heat capacity above ~ 400 °C is present only for thermal histories involving a rapid prior cooling, is reduced for faster heating rates, and is attributed to the exothermic decomposition of the super saturated solid solution produced upon rapid cooling. The 26 ng sample in **d** sees significant inconsistencies across different heating rates, though nevertheless follows the same trend of faster heating rates corresponding to a smaller apparent *c*_p_ drop. The upward inflexion of the C100H10k curves (red) before the onset of melting may be due to the dissolution of silicon that precipitated during the − 100 K s^−1^ cooling step, prior

The influence of cooling and heating rate on the measured heat capacity is shown in Fig. 5 for two sample sizes on two different sensors. Panels (a) and (c) show results on a 100 ng sample at a heating rate of 10,000 K s^−1^ after cooling at various rates and at varied heating rates after cooling at 30,000 K s^−1^, respectively. Panels (b) and (c) show similar plots for a 26 ng sample. The evaluation procedure to produce the curves is the same as for pure aluminium. Since fairly high consistency was found in the measurements on pure Al, and to avoid unnecessary measurements which age the chip sensor, the temperature programme was generally cycled twice. For measurements where less consistency was found between individual cycles, such as those involving lower heating or cooling rates, the temperature programme was repeated up to 8 times, from which the curves most consistent with the rest of the data were selected for averaging. The shorthand labels used in the figure legends refer to the programmed cooling and heating rates (“*β*_c_” and “*β*_h_” in Fig. 4); for example, “C1kH10k” denotes a cooling rate of − 1000 K s^−1^ and a subsequent heating rate of + 10,000 K s^−1^. The slow heating step at 1 K s^−1^ is the heat loss measurement and is a permanent feature of all temperature programmes.

An obvious feature of the curves in Fig. 5 is the depression in apparent heat capacity above ~ 400 °C. As expected, the 100 ng sample shows better consistency than the 26 ng sample. Importantly in (d), although not immediately clear from the plot, the same trend in apparent heat capacity depression is found as that seen in (c). The gradient variations seen in (c) and (d) both follow a trend of increasing gradient with slower heating rate. Beyond this, slower heating rates also have slightly higher melting onset temperatures. Finally, panels (a) and (b) see a slight upward inflexion shortly before melting for curves measured after the slowest cooling rate (C100 H10k, red).

**Fig. 6:**
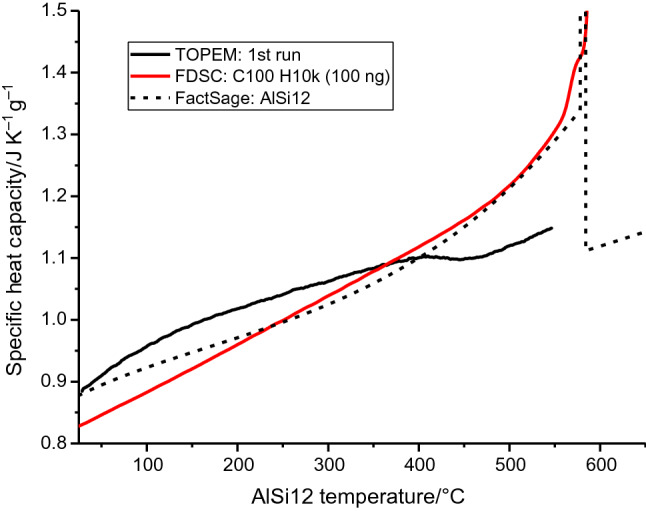
Heat capacity values are shown in context by comparing results from TOPEM and FDSC measurements of the AlSi12 powder to a FactSage calculation. The FactSage simulation well reflects the FDSC curve C100 H10k performed on the 100 ng sample

Finally, to provide some context and evaluate the accuracy and reliability of the collected FDSC data, Fig. 6 compares heat capacity curves from TOPEM measurements and a FactSage calculation for AlSi12 to the FDSC results. The “C100 H10k” curve of the 100 ng sample is included for comparison and is the FDSC measurement examined involving minimal Si precipitation. It correlates well to the simulated and TOPEM heat capacity curves. The TOPEM curve of the AlSi12 AM powder is a fair match at lower temperature, but shows a feature at 400–500 °C, possibly an indirect effect of the dissolution and precipitation occurring at that time.

## Discussion

The experimental approach first developed by examining high-purity aluminium is found to yield highly reproducible results over a wide span of scanning rates. This approach is then utilised for experiments on eutectic AlSi commonly used in additive manufacturing. Collecting precise data under non-equilibrium conditions are greatly relevant to AM, and the presented results on eutectic AlSi already reveal useful information that could directly impact AM process parameters or be fed into simulation calculations. Similar experiments for other materials could be easily derived from this same approach and promise a wealth of information on non-equilibrium material states and rapid-rate processes.

### * c*_p_ determination strategy

The results on pure Al presented in Fig. 1b, c, which depict a gradual drift to more endothermic heat flow values, tangibly demonstrate how such systematic errors generate an additional uncertainty in heat capacity. This phenomenon is noticeable for the high-temperature UFH 1 sensors and was not obvious with low-temperature UFS 1 sensors [9]. Since the melting enthalpy, sample colour and geometry do not change significantly during the experiments, and the sample is not altered by oxidation. Other possible effects could involve reaction between sensor and sample or changing sensor properties. The error of the heat capacity can be reduced by an improvement to experimental design as shown in Figs. 1a and 4. The principle of the improved determination method is to minimise the time between the segments which measure the heat loss and the sample heat flow. This minimises the impact of the observed incremental changes in absolute heat flow. The cycle’s cooling segments and fast heating segments can then be adjusted to suit individual measurement needs, and an approach which is followed in Fig. 4 for the subsequent measurements on AlSi12. Performing the slow-rate heat flow correction on individual pairs, that is, correcting measured heat flow by subtracting the preceding + 1 K s^−1^ heating scan from each fast heating scan should then yield more consistent results across multiple measurement cycles.

Further support for this measurement procedure is provided by Fig. 2, where the pair-wise heat flow correction (Fig. 2c) is shown to yield the best consistency.

The corrected heat capacity curves for pure Al collected in Fig. 3 cover the scanning rate range commonly utilised in FDSC scans and therefore, provide some insight into optimal measurement conditions. The inconsistencies found in the + 1000 K s^−1^ curves may be due to changes in the sample surface, the sample-membrane interface or some early stage fatigue of the chip’s sensors. Early stage aging of the sensor is certainly a possibility, crucially because those curves in Fig. 3a were measured before the other studied rates, and because the curves of Fig. 3a tend to approach those values of Fig. 3b–d with each successive cycle. This happens despite the execution of the standard sensor conditioning and the melting–solidification programmes. This last observation is of crucial importance, since it implies the sample sensor system does not reach a steady state after the initial melting cycles, but only after a few low-rate heating scans. Since this effect can impact the first few measurements on a sensor, it is worth considering in experimental design.

Since the determined *c*_p_ curves are all ~ 10% lower than the literature values, the 64 ng mass determined from the pure Al sample’s melting peak is suspected to be ~ 10% higher than the true sample mass. Other than this apparent systematic error, the curves are highly consistent and parallel to the expected values; as such, similar heat capacity measurements could be a useful tool for assessing the accuracy of the FDSC determined mass. A similar issue of apparent mass inaccuracy was also found for pure Pb with UFS1 chip sensors [9]. Although in that case the *c*_p_ was always overestimated (meaning calculated mass was too low) rather than underestimated as in the present case for Al on the UFH1 chip sensors. In both cases, the results benefit from knowing the material’s equilibrium heat capacity; however, precise mass estimation remains a source of probable inaccuracy for *c*_p_ calculation in FDSC experiments [8].

In any case, the results presented in Fig. 3 represent a successful adaptation of the experimental design of [9] to the specific requirements of UFH 1 chip sensors; after some initial changes in the measurement values, the results converge upon the literature heat capacity and exhibit excellent reproducibility, establishing this pair-wise heat flow correction as a sound method.

### Application to AlSi12 AM powder

The developed method for heat capacity determination is generalised schematically in Fig. 4. This experimental approach is followed for the heat capacity measurements presented in Figs. 3 and 5 and may also serve as a template for precise heat flow measurements by FDSC on other materials. The cooling rates for the experiments on AlSi12 were chosen in the range from near-equilibrium to strong, non-equilibrium conditions. Tuning the maximum and minimum temperatures, as well as the programmed fast heating and cooling rates (*β*_h_ and *β*_c_ in Fig. 4), to suit the individual needs of the material is a valid approach for precise heat flow measurements in general, provided the system in question is suited to cyclic analysis via the slow-rate heat flow correction method. That is, providing the sample properties of interest are not irreversibly changed during the slow heating segment. In general, metallic systems are well suited to this approach, since the system properties investigated can be consistently reproduced by cooling from the molten state. Taking a similar approach on other alloy systems promises a wealth of useful data.

Regarding the present results on AlSi12, Fig. 5 shows a clear heat capacity depression above ~ 400 °C dependent on the chosen heating and cooling rates. The measured apparent heat capacity curves displayed in Fig. 5 are the sum of (i) the sensible specific heat capacity and (ii) the latent specific heat capacity due to any thermal effect occurring during heating. The curves in Fig. 5 therefore show that a significant exothermic event occurs on heating above ~ 400 °C. This event increases with a more rapid previous cooling rate. Assuming that faster cooling produces a stronger supersaturation, which has higher propensity for precipitation, points to precipitation causing the release of heat and the depression in determined apparent heat capacity. This is corroborated in the literature, where Si precipitation is reported at temperatures as low as 135 °C for a similarly composed AlSi alloy [43], whilst many other studies into AlSi systems show Si precipitation as a surety when reheating rapidly cooled material [25, 44].

Looking then at panels (c) and (d) with constant − 30,000 K s^−1^ prior cooling show the curves measured at lower heating rates have a larger precipitation event. This aligns with expectations, since during slow heating the sample spends more time at precipitation possible temperatures before melting at ~ 585 °C. This melting temperature is slightly higher than the reported eutectic temperature of 577 °C [45], likely a result of some device temperature inaccuracy. For the slowest prior cooling rate, an upward inflexion is seen prior to melting. This could be associated with the endothermic equilibrium dissolution of Si, known from the solubility of Si in Al [45], and also seen in the calculated apparent *c*_*p*_ curve from FactSage in Fig. 6. Additionally, the impact of heating rate on melting temperature, though minor, is possibly a consequence of grain growth/coarsening during heating, since smaller grains cause lower melting temperatures, and vice versa, larger grains comparatively higher melting temperatures [46, 47]. Much of the success of AlSi12 and AlSi10Mg for LPBF is attributed to the presence of silicon [27], since it is largely responsible for heat absorption from the scanning laser [48], and also because of its impact on solidification by helping to reduce solidification cracking. Cracking during solidification is related to the solidification range of the alloy, the undercooling [20], the fluidity of the molten phase, the solidification shrinkage and the coefficient of thermal expansion; parameters which are all improved by a near-eutectic silicon content [27]. A deepened understanding of phase content during rapid processing is therefore highly relevant to AM.

The comparison of TOPEM, FDSC and simulated heat capacity curves in Fig. 6 shows decent consistency across the three methods and establishes good confidence in the accuracy of the FDSC results. The “C100 H10k” measurement is chosen for comparison here since it involves little precipitation. The *c*_p_ depression seen in the TOPEM result occurs at the same temperature precipitation is found in the FDSC data, suggesting the precipitation and dissolution there as at least an indirect cause. Equilibrium *c*_p_ measurements of the as-produced AM powder, which is far from equilibrium, may not be the most optimal approach, even using temperature modulation. Tuning of parameters such as temperature amplitude could provide some benefit in this regard, but are outside of the scope of this work.

A broad assessment of the collected measurements on all three sensors also reveals some basic information regarding optimal measurement parameters. For the measurement of Al-based materials, the optimum sample size for the UFH 1 sensor seems to be between 50 ng and perhaps a few hundred nanograms. This is supported by the present results since the lowest mass of 26 ng showed some inconsistency, particularly at heating rates below + 10,000 K s^−1^ (see Fig. 5d); although the specific cause of this is not certain, the low mass seems likely. The 100 ng and 67 ng samples both performed well, with the exception of some noise on heating at + 1000 K s^−1^ (see Fig. 3a). Measurements at higher rates benefit from a better signal-to-noise ratio, with the noise at + 5000 to + 30,000 K s^−1^ having little impact on the determined curves, though the thermal lag at the highest rates can impact the range of usable results. Since high consistency was generally found for the studied samples, 2–6 measurements were sufficient for averaging. These general observations could serve to inform future experiments on UFH 1 sensors, particularly for metallic materials.

Whilst the phenomena of Si precipitation in quenched AlSi alloys is certainly nothing new, its impact on apparent heat capacity at such high heating rates, as found in LPBF, is very valuable data. Contemporary modelling and simulation studies in AM often rely on fixed or equilibrium values of heat capacity [49–52] and could certainly benefit from improved data here. Depending on the actual thermal history, apparent heat capacity could be only 35% of the equilibrium value (Fig. 5). Moreover, the precipitation observed in the heat capacity data could have direct implications on the process parameters for AM and on the understanding of how microstructure and phase composition evolve during printing. The work of Yang et al. on the microstructure of single AlSi12 powder particles dependent on particle size and undercooling is also highly relevant here [21]. The precipitation revealed in Fig. 5 goes some way to understanding how post-solidification heat spikes might impact the evolving microstructure, and how absorptivity and heat transfer might evolve during the process; considerations which dictate the optimal process parameters like laser power, scan speed and hash spacing.

## Conclusions

Using high-purity aluminium as a standard for assessing accuracy and precision, a measurement strategy based on a slow-rate heat flow correction is developed and refined for specific heat capacity measurements in fast differential scanning calorimetry using MultiSTAR UFH 1 chip sensors. This strategy is then applied to the study of the aluminium alloy AlSi12, with the aim of further understanding the kinetic impacts and microstructural changes caused by high heating and cooling rates, and the results are discussed in the context of metal additive manufacturing. FactSage thermodynamic simulations and temperature-modulated DSC measurements contextualise the FDSC results on AlSi12. Differences in the measured apparent heat capacity reveal strong decomposition effects of super saturated solid solution, which are highly relevant to processing and for understanding the microstructure and phase content evolution during additive manufacturing methods such as laser powder bed fusion. The measurement method herein developed provides a reliable method for precision heat flow measurements involving rapid heating and cooling via FDSC and can be easily adapted to suit many materials, especially metals and metal alloys used for additive manufacturing.
